# *Bacteroides* abundance drives birth mode dependent infant gut microbiota developmental trajectories

**DOI:** 10.3389/fmicb.2022.953475

**Published:** 2022-10-06

**Authors:** Dollwin Matharu, Alise J. Ponsero, Evgenia Dikareva, Katri Korpela, Kaija-Leena Kolho, Willem M. de Vos, Anne Salonen

**Affiliations:** ^1^Human Microbiome Research Program, Faculty of Medicine, University of Helsinki, Helsinki, Finland; ^2^Department of Biosystems Engineering and BIO5 Institute, University of Arizona, Tucson, AZ, United States; ^3^Children's Hospital, Pediatric Research Center, University of Helsinki and HUS, Helsinki, Finland; ^4^Faculty of Medicine and Health Technology, University of Tampere, Tampere, Finland; ^5^Laboratory of Microbiology, Wageningen University, Wageningen, Netherlands

**Keywords:** *Bacteroides*, shotgun metagenomics, infant gut microbiota, functional maturation, hierarchical clustering, vaginal delivery, cesarean section

## Abstract

**Background and aims:**

Birth mode and other early life factors affect a newborn's microbial colonization with potential long-term health effects. Individual variations in early life gut microbiota development, especially their effects on the functional repertoire of microbiota, are still poorly characterized. This study aims to provide new insights into the gut microbiome developmental trajectories during the first year of life.

**Methods:**

Our study comprised 78 term infants sampled at 3 weeks, 3 months, 6 months, and 12 months (*n* = 280 total samples), and their mothers were sampled in late pregnancy (*n* = 50). Fecal DNA was subjected to shotgun metagenomic sequencing. Infant samples were studied for taxonomic and functional maturation, and maternal microbiota was used as a reference. Hierarchical clustering on taxonomic profiles was used to identify the main microbiota developmental trajectories in the infants, and their associations with perinatal and postnatal factors were assessed.

**Results:**

In line with previous studies, infant microbiota composition showed increased alpha diversity and decreased beta diversity by age, converging toward an adult-like profile. However, we did not observe an increase in functional alpha diversity, which was stable and comparable with the mother samples throughout all the sampling points. Using a *de novo* clustering approach, two main infant microbiota clusters driven by *Bacteroidaceae* and *Clostridiaceae* emerged at each time point. The clusters were associated with birth mode and their functions differed mainly in terms of biosynthetic and carbohydrate degradation pathways, some of which consistently differed between the clusters for all the time points. The longitudinal analysis indicated three main microbiota developmental trajectories, with the majority of the infants retaining their characteristic cluster until 1 year. As many as 40% of vaginally delivered infants were grouped with infants delivered by C-section due to their clear and persistent depletion in *Bacteroides*. Intrapartum antibiotics, any perinatal or postnatal factors, maternal microbiota composition, or other maternal factors did not explain the depletion in *Bacteroides* in the subset of vaginally born infants.

**Conclusion:**

Our study provides an enhanced understanding of the compositional and functional early life gut microbiota trajectories, opening avenues for investigating elusive causes that influence non-typical microbiota development.

## Introduction

The acquisition and development of early life gut microbiota have been linked to health outcomes during infancy and later life (Avershina et al., 2021; Sarkar et al., 2021). The establishment of gut microbiota is affected by several maternal factors, perinatal and postnatal exposures such as mode of birth, breastfeeding, infections, antibiotics, hosts genetics, and living environment (Penders et al., 2006; Tamburini et al., 2016; Korpela and de Vos, 2018). Delivery mode is a crucial factor especially in the first months of life (Arboleya et al., 2018; Kumbhare et al., 2019; Korpela, 2021). As infants develop, their microbiota matures through a phase characterized by high dynamics and low diversity in early infancy (from birth to 6 months) and reaches a more stable and diverse microbial community during early childhood (3–5 years old) (Milani et al., 2017; Korpela and de Vos, 2018; Korpela, 2021). Importantly, the maturation of infant gut microbiota is affected by the timing of weaning and breastfeeding cessation, as these events contribute to shifting of the microbial community composition toward an adult-like profile (Bäckhed et al., 2015).

Several authors have reported the critical impact of delivery mode on early-life microbiota acquisition. Typically, Cesarean-section delivered (CSD) infants show a strong depletion in *Bacteroides* that typically resolves between 6 and 12 months of age in relation to weaning (Shao et al., 2019; Yang et al., 2019; Korpela, 2021; Princisval et al., 2021). Some studies suggest that birth mode-related impact on early life microbiota may take 3–5 years to normalize (Roswall et al., 2021). On the other hand, vaginally delivered (VD) infants' gut microbiota is typically dominated by *Bifidobacterium* and *Bacteroides spp*.; both genera are known to be central in digestion of human milk oligosaccharides (Marcobal et al., 2010). The CS birth-related scarcity of *Bacteroides spp*. has been linked to increased risk of intestinal colonization by opportunistic pathogens (Reyman et al., 2019; Shao et al., 2019) and altered immunostimulatory potential in infants (Wampach et al., 2018). Overall, *Bacteroides spp*. have been suggested to play a pivotal role in infant health because of their role in modulating and training the immune system (Vatanen et al., 2016), and higher ratio of *Enterobacteriaceae* to *Bacteroidaceae* in CSD infants has been implicated in increased risk of development of atopy and *Clostridioides difficile* infection in late infancy (Vu et al., 2021). Some species of *Bacteroides* are involved in cross-feeding nutrients with other gut microbes, producing antimicrobial toxins and competing against pathogens (Wexler, 2007; Zafar and Saier, 2021). The lack of exposure to mother's gut microbiota at birth explains the delayed establishment of *Bacteroides* spp. in CSD babies (Korpela et al., 2020; Korpela, 2021). However, some VD infants are also essentially devoid of *Bacteroides* spp. (Yassour et al., 2016; Shao et al., 2019; Wilson et al., 2021). Characterization of individual variations influencing the abundance of *Bacteroides* spp. in the infant gut will contribute to the enhanced understanding of the physiological role of this key early life colonizer.

Most studies to date have described the dynamics of infant gut microbiota development through 16S rRNA gene amplicon sequencing approaches. When combined with unsupervised clustering approaches, these studies have provided important insights into temporal, ethnicity related, and age-specific development of microbiota trajectories as well as the stability, inferred functions, and maturation rates of community types (Stewart et al., 2018; Borewicz et al., 2019; Roswall et al., 2021; Tun et al., 2021; Xiao et al., 2021). Since amplicon-based studies restrict exploring the functional capacities of microbial communities, much less is known about the functional aspects of infant microbiota development. A recent Canadian study based on the well-characterized CHILD birth cohort used clustering approaches and conducted metagenomic imputation to provide novel insights into the potential role of infant's gut microbiota-mediated sphingolipid metabolism in food sensitization, linking *Bacteroides*-depleted microbiota clusters to higher odds of developing atopic sensitization in a host genotype-dependent manner (Tun et al., 2021). A recent meta-analysis of multiethnic infant microbiota enterotypes also characterized the functional aspects of microbiota maturation during the first years and showed that *Bacteroides-* and *Prevotella-*dominated infant enterotypes share enriched metabolic pathways, e.g., glycolysis and starch degradation (Xiao et al., 2021). Earlier metagenome studies have provided important insights into the temporal development of gut microbiota mainly in VD infants (Bäckhed et al., 2015; Stewart et al., 2018; Busi et al., 2021). However, the anticipated impact of delivery mode on the functional capacities of infant gut microbiota is still not fully understood. A recent metagenome study reported that the characteristic signature of low *Bacteroides* abundance in CSD infants led to the underrepresentation of several biosynthetic pathways at the age of 3 months (Wilson et al., 2021). Our study leverages the shotgun metagenomic sequencing approach to explore the dynamics of infant gut microbiota development. This study comprises samples spanning 3 weeks to 1 year of age in a group of 78 term infants from the well-characterized Finnish Health and Early Life Microbiota (HELMi) birth cohort (Korpela et al., 2019). Using a clustering approach, we defined three main microbiota development trajectories differing in both taxonomic and functional compositions. While the trajectories were strongly dependent on delivery mode, we identified a group of VD infants presenting depletion in *Bacteroides* spp. comparable to that in CSD infants. We further characterized this unusual microbiota composition for VD infants and investigated the underlying factors as well its impacts on the functional capabilities of the microbiota.

## Materials and methods

### Sample collection and sequencing

The HELMi birth cohort study (*N* = 1,055) is a prospective follow-up study on early life microbiota and health (Korpela et al., 2019). Healthy infants born on gestational weeks 37–42 without known congenital defects and exceeding the birth weight of 2.5 kg were included in the cohort. For this study, 90 infants representative of key early exposures (N = 25 born by CS, N = 24 VD with intrapartum antibiotics (IAP), and N = 41 VD without IAP) were selected from the HELMi cohort. The infants were selected from the broader HELMi cohort based on the number of samples available and their birth mode. Infants who had a long-term non-allergic disease reported by the age of 2 years were excluded. All CS deliveries involved IAP, and among the CSD infants, 14 were born by planned CS and 11 were by emergency CS. No restrictions in terms of maternal health status or maternal antibiotic treatment during the pregnancy period were applied in selecting the infants for this study.

Fecal samples were collected at the age of 3 weeks and 3, 6, and 12 months, and their mothers' samples were collected within 2 weeks prior to childbirth and were subjected to shotgun metagenome sequencing. The study was approved by the ethical committee of the Hospital District of Helsinki and Uusimaa and performed in accordance with the principles of the Helsinki Declaration. The parents signed informed consent for enrolment. They collected maternal and infant fecal samples and stored them at home in freezers at −20°C. The samples were transported frozen to the study center within 6 months of collection and were stored in freezers at −80°C upon arrival. Information on early life exposures was collected through online questionnaires and hospital records. The infants were generally healthy, although two were diagnosed with an allergic disease by the age of 1 year.

Fecal DNA was extracted from fecal material (0.026 to 2.46 g, median = 0.266 g) by suspension in 1 ml of sterile ice-cold PBS, and 250 μl of the fecal suspension was combined with 340 μl of RBB lysis buffer (500 mM NaCl, 50 mM Tris-HCl (pH 8), 50 mM EDTA, and 4% SDS) in a bead-beating tube from Ambion Magmax™ Total Nucleic Acid Isolation Kit (Life Technologies, Carlsbad, CA, United States). After repeat bead-beating, 200 μl of the supernatant was used for DNA extraction with a KingFisherTM Flex automated purification system (Thermo Fisher Scientific, Waltham, MA, United States) using MagMAXTM Pathogen High Vol. DNA was quantified using the Quanti-iT™ Pico Green dsDNA Assay (Invitrogen, San Diego, CA, United States). Sequencing libraries were prepared according to the Nextera DNA Flex Library Prep Reference Guide (v07) (Illumina, San Diego, CA, United States), with the exception that the reaction volumes were scaled down to $\frac{1}{4}$ of the protocol volumes. Sequencing was performed with an Illumina NovaSeq system using S4 flow cells with a lane divider (Illumina, San Diego, CA, United States) at the sequencing laboratory of the Institute for Molecular Medicine Finland (FIMM) Technology Center, University of Helsinki. Each pool was sequenced in a single lane. The read length for the paired-end run was 2 × 151 bp.

### Quality control and human read filtering

Quality filtering and removal of human sequences were performed on Fastq files from NovaSeq using fastqc v0.11.9 and trimGalore v0.6.6 with default parameters. Quality-filtered sequences were screened to remove human sequences using bowtie2 v2.4.2 against a non-redundant version of the Genome Reference Consortium Human Build 38, patch release 7 (available at https://genome-idx.s3.amazonaws.com/bt/GRCh38_noalt_as.zip). After quality control and human read filtering, metagenomes at the early time points (3 and 12 weeks) containing < 10 million paired-end reads were discarded based on the observed drop in species richness in shallow sequenced samples. For the later time points (6 and 12 months) and mother samples, metagenomes with < 20 million paired-end reads were discarded. Infants with < 3 samples remaining were excluded from the analysis. Overall, 12 infants (*N* = 2 born by CS, *N* = 9 VD delivery, and *N* = 1 VD with IAP) were excluded because of low sequencing depth or insufficient number of samples. A total of 78 infant samples were kept for further analysis.

### Taxonomic and functional profiling

Taxonomic profiling of the metagenomic samples was performed using Kraken2 (Wood et al., 2019) and Braken (Lu et al., 2017). Briefly, Kraken2 v2.1.1 was run on the paired read using the HumGut database (Hiseni et al., 2021), and Bracken v2.6.1 was run on Kraken2 outputs. Functional profiling was realized on the quality-controlled and human-filtered reads using HumaNn 3.0 (Beghini et al., 2021). The translated search was carried out using the UniRef90 database and hits were mapped to their corresponding Kyoto Encyclopedia of Genes and Genomes (KEGG) Orthogroups (KOs). Gene family counts were normalized as count per million reads before the analysis. Gene counts were mapped to MetaCyc pathway definitions, and MinPath was used to calculate pathway abundances.

Alpha diversity measure (Shannon index), richness (Chao1 index), and beta diversity distances between samples (Bray-Curtis) for the taxonomic and functional profiles were estimated with the R packages *vegan* (Dixon, 2003) and *microbiome* (Lahti and Shetty, 2012-2019). The stability of bacterial composition during growth was explored by calculating the presence-absence Jaccard index for successive time points for each child. The Jaccard index was computed on the relative abundances at the genus level using the package *vegan*.

### kmer-composition profiling

Simka v1.5.3 (Benoit et al., 2016) was run on metagenomic datasets with the following parameters: -abundance-min 2 -max-reads [MINCOUNT] -simple-dist, where [MINCOUNT] is the smallest sequence count across the analyzed samples. A Bray-Curtis distance computed on the content in the kmer of metagenomes was obtained and used for further analysis.

### Hierarchical clustering of the samples based on taxonomic composition

The compositional analyses were performed using R (version 3.4.4). First, the taxonomy data were aggregated at the family level. Low-abundance families (< 1% relative abundance and < 0.5% prevalence) were filtered out, and zero values were replaced by an estimate of 1. Then, the dataset was normalized by taking the centered log ratio (CLR), and a distance matrix was calculated from the transformed data using the Euclidean distance (Aitchison distance) (Aitchison et al., 2000; Tsilimigras and Fodor, 2016). Hierarchical clustering was carried out separately for each time point with the function *hclust* and with the Wald.D2 method. To determine the appropriate number of clusters, the silhouette coefficient was calculated and the number of clusters allowing the highest silhouette coefficient was selected. The significance of the obtained clusters was confirmed by PERMANOVA at the family level using the Aitchinson distance. The association of the sample clusters to exposures such as intrapartum and postnatal antibiotics was assessed by Fisher's exact test and was not found to be significant at any time point.

### Defining VD high- and low-*Bacteroides* groups

The VD infants were grouped as “*Bacteroides*-present VD” and “*Bacteroides*-depleted VD” according to the relative abundance of the *Bacteroides* genus in the samples collected at 3 weeks. A cut-off of 1% relative abundance at the genus-level was used to define the groups. The 1% cut-off was chosen as all the CS infants had a relative abundance of *Bacteroides* below this threshold. The association of the infant groups with IAP and postnatal antibiotic exposure was assessed by Fisher's exact test and was found to be non-significant.

### MaAsLin2 differential abundance analysis

To assess the associations between infant microbiota clusters at each time point and MetaCyc pathway abundances, pathways with at least 0.0001% relative abundance in at least 10% of the samples were selected. The associations between pathways and clusters were determined with generalized linear and mixed models, using Multivariate Association with Linear Models (MaAsLin2) (Mallick et al., 2021). Any pathway association with an FDR- adjusted *p*-value < 0.1 was considered statistically significant.

Associations between maternal microbiota composition and the grouping of VD infants according to their relative abundance of *Bacteroides* were assessed using the Negative Binomial model from the MaAsLin2 package (-m NEGBIN parameter). Taxonomic profiles were aggregated at the species level, low abundance hits were filtered out (detection below 0.001% and prevalence below 1%), and counts were normalized by TMM (Trimmed Mean by M-Values). The results obtained through negative binomial models were confirmed using generalized linear models from the MaAsLin2 package. Any species association with an FDR-adjusted *p*-value < 0.1 was considered statistically significant.

### Association between infant microbiota and environmental variables

A directed acyclic graph (DAG) was built detailing causal relationships affecting the association between perinatal and postnatal exposures and infant gut microbiota development (Supplementary Figure 1). Nodes represent the exposure of interest, and each node is interrelated by directional arrows that represent theoretical associations based on the researchers' assessment of previous literature and confirmed by association testing in the HELMi cohort. From this representation, we assessed that birth mode is a potential confounder of the effect of perinatal variables (intrapartum antibiotics, gestational age, time of first skin-to-skin contact, and number of previous deliveries) and breastfeeding variables (length of breastfeeding time, length of exclusive breastfeeding, and age of first solids). Tests of associations between these variables and the infant gut microbiota were therefore controlled for birth mode effects.

For testing the association between infant microbiota trajectories and environmental variables (Results Section Taxonomic and functional features of the birth mode-driven infant microbiota clusters) as well as between the VD *Bacteroides* infant group and environmental variables (Results Section CS-like *Bacteroides* depletion in early life microbiota can be observed in a subset of VD infants), a large number of tests were carried out in an attempt to identify any variables that may be significantly associated to a group of samples. An FDR correction was applied to account for the increased risk of Type I error in multiple testing settings, and FDR adjusted *p-*values < 0.1 were considered statistically significant.

A *post-hoc* sensitivity analysis was conducted with the G^*^Power software version 3.1.9.2 to retrospectively examine the observed power of the study. The effect size required for a power level of 0.8 was calculated with the number of samples used at the significance level (*p* = 0.05) for the Wilcoxon–Mann–Whitney test.

### Other statistical analysis

The statistical analysis was conducted using R (version 3.4.4) with the packages *vegan* and *microbiome* (Dixon, 2003; Lahti and Shetty, 2012-2019). Alluvial diagrams were constructed with the R package ggalluvial (Brunson, 2020).

For univariate data comparison, a statistically significant difference was evaluated by the unpaired Wilcoxon test when applied to numeric variables or the Fisher test when comparing categorical groups, and *p*-values < 0.05 were considered statistically significant. Additionally, when a pair-wise Wilcoxon test was carried out on more than two groups of samples, an FDR correction was applied, and FDR adjusted *p*-values < 0.05 were considered statistically significant.

## Results

### Cohort overview

The present study included 78 infants from the HELMi cohort [NCT03996304 (Korpela et al., 2019)], born in Finland at hospitals at term, and followed up for 1 year. A total of 55 infants were born *via* VD. All the 23 CS deliveries and 23 VDs involved IAP. Baseline characteristics and antibiotic exposures over the first year of life for all 78 infants, stratified by mode of delivery and IAP exposure, are summarized in Supplementary Table 1. Perinatal and background variables were evenly distributed over the three birth groups except for two environmental exposures (siblings and pets at home) and antibiotic exposures during the first year of life (Supplementary Table 1). All the children were breastfed during the first months of life. Most of the children were breastfed for more than 9 months, and only 8 were breastfed for 6 months or less. The introduction of solid food took place between 18 and 31 weeks of age. Seventeen (22%) children received at least one antibiotic treatment during their first year of life, and some received multiple courses (30 courses in total for all the children) (Supplementary Table 1). Infants' gut microbiota was assessed by shotgun metagenomic sequencing of fecal samples collected at 3 weeks (*n* = 75) and 3 months (*n* = 77), 6 months (*n* = 71), and 1 year (*n*=57). Each infant was sampled a minimum of 3 times, with 60% of the infants (*n* = 46) having all 4 samples. For 50 infants, metagenomic data were also available from their mother's stool samples taken during the last 2 weeks before delivery.

### Microbiota maturation over the first year of life

**Figure 1:**
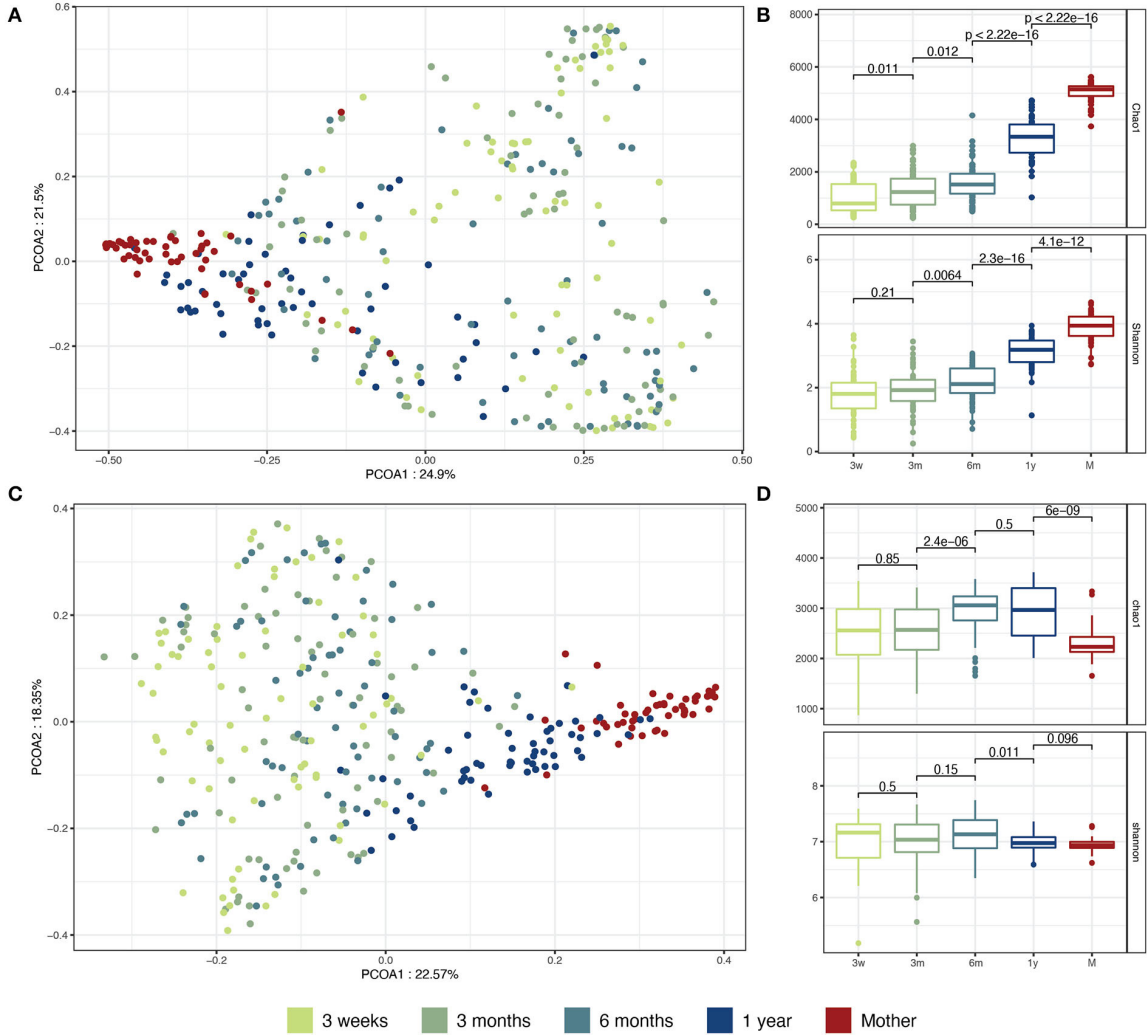
Taxonomic and functional maturation of infant microbiota during the first year of life. **(A)** PCoA on genus level taxonomic profiles. Taxonomic profiles were aggregated at the genus level. The beta diversity Bray-Curtis distance was computed between sample pairs and plotted as a PCoA. **(B)** Taxonomic alpha diversity and richness. Taxonomic profiles were aggregated at the species level. The Shannon alpha-diversity index and the Chao1 richness index were computed for each sample. **(C)** PCoA on KO functional profiles. The KO profiles were normalized as count per million reads. The beta diversity Bray-Curtis distance was computed between each sample pair and plotted as a PCoA. **(D)** Functional alpha diversity and richness. The KO profiles were normalized as previously described. The Shannon alpha-diversity index and the Chao1 richness index were computed for each sample. The comparisons between groups were conducted by unpaired Wilcoxon test.

After quality filtering and removing human sequences, the metagenomes had an average of 39 million paired-end reads per sample. These high-quality, human-filtered reads were subjected to taxonomic annotation with Kraken2 (Wood et al., 2019) using the HumGut database (Hiseni et al., 2021). The taxonomic assignment obtained with Kraken2 was recalculated using Braken (Lu et al., 2017). This method has been previously shown to achieve high taxonomic precision for gut taxonomic profiling (Tamames et al., 2019; Ye et al., 2019; Allnutt et al., 2021). On average, 91% of the total reads at the phylum level and 77% at the family level were successfully annotated. In the infant samples, the most abundant bacterial families were *Enterobacteriaceae* (27% global average relative abundance), *Bifidobacteriaceae* (22%), *Bacteroidaceae* (15%), *Lachnospiraceae* (7%), *Veillonellaceae* (4%), *Clostridiaceae* (4%), *Oscillospiraceae* (4%), and *Streptococcaceae* (1%). Other families represented in total 14% of the sequences (Supplementary Figure 2). The infants' microbiota maturation was characterized by a global decrease in interindividual beta diversity and convergence toward an adult-like profile (Figure 1A) and had a progressive increase in *Lachnospiraceae* and *Oscillospiraceae* during the first 12 months (Supplementary Figure 2). However, at 1 year, the infants' gut microbiota composition was still significantly different from that of the mothers' microbiota (PERMANOVA on genus-level Aitchinson distance, *p* = 0.001; 999 permutations). The alpha diversity (Shannon diversity index) did not significantly increase between 3 weeks and 3 months but significantly increased between 3 and 6 months (Wilcoxon *p* < 0.05) (Figure 1B), coinciding with the age when solid foods were introduced to the children's diet (median = 22 weeks in the cohort). Significant differences in alpha diversity were also observed between 6 and 12 months, and between 12 months and adult microbiota. Similarly, species richness (Chao1 index) increased during infancy, especially between 6 and 12 months, but was significantly higher in the adult samples than in the 12-month samples (Wilcoxon *p* < 0.05) (Figure 1B). The results were similar after rarefying the samples to the same sequencing depth (data not shown) (Bäckhed et al., 2015).

We also analyzed gut microbiota maturation using an annotation-free approach that computes distances between microbial communities directly based on the number and abundance of shared words of length *k* (k-mers) (Benoit et al., 2016). Using this approach, we observed a global maturation of the infant samples toward an adult-like sequence composition (Supplementary Figure 3A) and a significant decrease in the inter-individual beta diversity during growth. However, no interindividual beta diversity decrease was observed between the 12-month samples and the mother samples (Supplemental Figure 3B). This could be explained by the increased heterogeneity in subspecies diversity in the adult samples.

Next, we explored how the maturation of the microbiota affected the microbial metabolic and functional pathways. KEGG Orthogroup (KO) counts were obtained for all the samples and mapped to the main KEGG cellular metabolic pathways. KOs involved in amino acid metabolism, carbohydrate metabolism, and energy metabolism were found to be the most abundant at all infant ages and in the mother samples (Supplementary Figure 4B). Similar to the taxonomic maturation, we observed functional maturation of the infant microbiota, with the infant samples slowly converging toward an adult-like composition during the first year of life (Figure 1C). The functional alpha diversity (Shannon diversity index on KO counts) was globally stable during the first year of life and comparable between the infant and mother samples. On the other hand, the gene richness (Chao1 index on KO counts) significantly increased in infants between 3 and 6 months but was surprisingly found to be lower in the mother samples (Figure 1D). To control for potential biases in the functional annotation as an explanation for the decreasing trend of gene richness, we looked at the proportion of sequences left out of functional annotation. Indeed, the proportion of sequences unmapped to a gene family increased significantly between 6 and 12 months in the infants and was highest in the mother samples (Supplementary Figure 4A), suggesting increased complexity of the microbiome in late infancy and further in adulthood.

### Taxonomic and functional features of the birth mode-driven infant microbiota clusters

**Figure 2:**
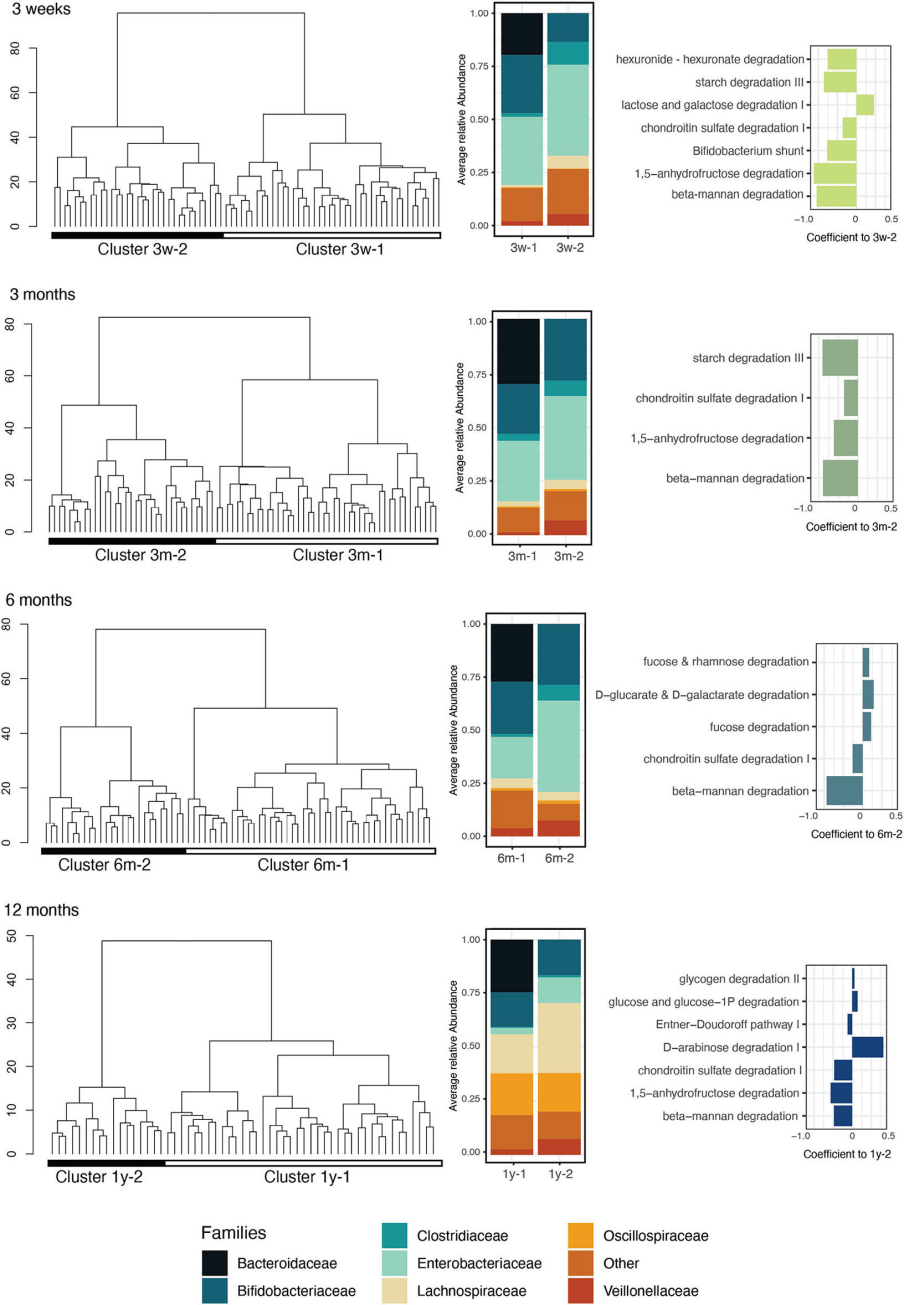
Infant gut microbiota clusters, representative bacterial families, and enriched functions related to carbohydrate metabolism. At each sampling time point (3 weeks, 3 months, 6 months, and 1 year), the infants' samples were clustered by hierarchical clustering on taxonomic profiles aggregated at the family level using the Aitchison distance (Column 1). The color blocks below the dendrograms represent the clusters identified using the silhouette maximization method (Column 2). The average relative abundance of the families in each cluster was represented in the associated bar plots. Families with detection and prevalence below 10% were grouped into the “Other” category (Column 3). Bar plot showing the coefficients of differentially abundant carbohydrate metabolism-related pathways (MaAsLin2, linear model, *q* < 0.1) in the two identified clusters. Negative coefficients indicate association to cluster 1.

To identify the main microbiota establishment trajectories, we clustered the infant samples on their taxonomic composition at the family level for each sampling time point. We performed hierarchical clustering of the samples using the ward linkage on the Aitchinson distance. The bacterial communities clustered into two groups at each sampling time point (Figure 2). The clusters were confirmed by PERMANOVA and showed that the composition of the samples was significantly different between clusters at the family level at each time point (PERMANOVA on family-level Aitchinson distance, *p* = 0.001; 999 permutations). However, the alpha diversity was not significantly different between the clusters, and their taxonomic richness was significantly distinct between the clusters only in the 3-week and 12-month samples (Wilcoxon *p* < 0.05) (Supplementary Figure 5), suggesting that the identified clusters differed in terms of composition but not in terms of taxonomic complexity. The PCA bi-plot for the clusters revealed that the clustering was driven at all time points by the abundance of the *Bacteroidaceae, Tannerallaceae, Rikennellaceae*, and *Ordoribacteriaceae* families in the first cluster, and the presence of *Clostridiaceae* in the second (Supplementary Figure 6). The cluster membership was strongly associated with the birth mode at all time points (Fisher's exact test *p* < 0.05) but was not associated with IAP exposure (Fisher's exact test *p* > 0.05 between VD and VD + IAP infants) or postnatal antibiotic exposure (Fisher's exact test *p* > 0.05).

We next compared the identified clusters in terms of their functional composition. At all the time points, the clusters had a significantly different functional composition (PERMANOVA on KO Bray-Curtis distance, *p* = 0.001; 999 permutations). The KO alpha diversity (Shannon index) was not significantly distinct between the clusters at any sampling time point; however, the KO richness (Chao1 index) was found to be significantly lower in cluster 2 at 3 weeks (cluster 3w-2) (Supplementary Figure 5). To provide an overview of the variability patterns of microbiota functions between the clusters, we mapped the gene families to 401 prokaryotic MetaCyc pathways and identified differentially abundant pathways between the clusters for each time point. At 3 weeks, 49 pathways were identified as significantly differentially abundant in the two clusters; at 3 months, 55 pathways; at 6 months, 73 pathways and at 12 months, 120 pathways were found significantly differentially abundant between the clusters (linear model, *q* < 0.1). Across all the time points, in total, 68 unique pathways were enriched in clusters 3w-2, 3m-2, 6m-2, and 1y-2, in particular pathways involved in nucleotide biosynthesis (18% of the differentially abundant pathways), carbohydrate biosynthesis (13%), and cofactor biosynthesis (10%). On the other hand, 124 pathways were enriched in clusters 3w-1, 3m-1, 6m-1, and 1y-1, in particular pathways involved in amino acid biosynthesis (15%), cofactor biosynthesis (14%), and carboxylate degradation (8%) (Supplementary File 1). Pathways involved in carbohydrate degradation were consistently differentially abundant between the two clusters at all the time points and were mostly associated with *Bacteroidaceae*-driven cluster 1, especially in the early time points (Figure 2). Interestingly, pathways involved in the degradation of beta-mannan, a plant-based dietary polysaccharide, were consistently enriched in this cluster throughout the first year and already before weaning age (Figure 2). The complete list of identified pathways and their differences between the clusters are listed in Supplementary File 1.

**Figure 3:**
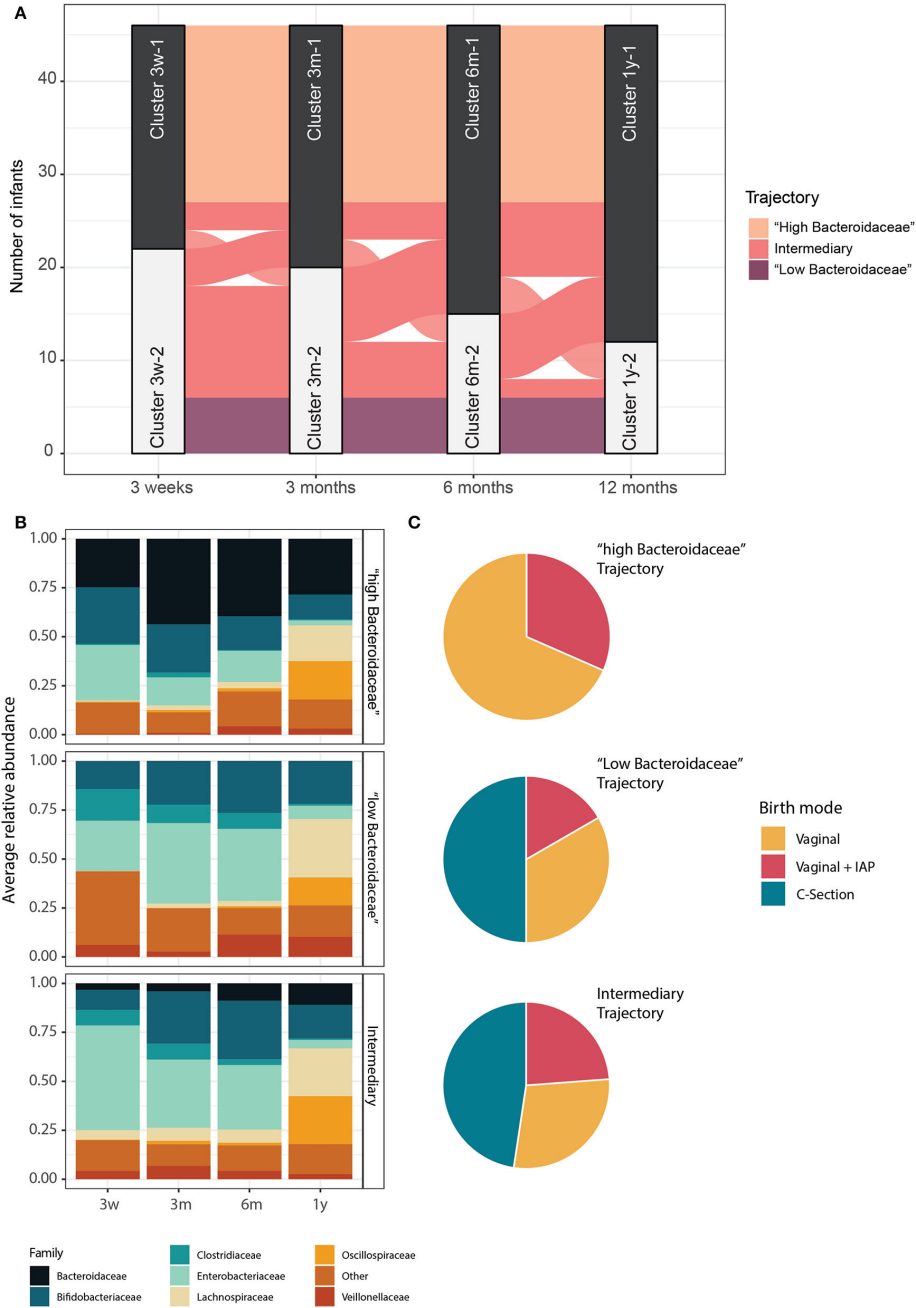
Three major microbiota development trajectories identified. **(A)** Alluvial plot showing the evolution of infants' microbiota trajectories through the first year of life, grouped according to the microbiota clusters identified at each time point. Groups of infants are colored according to the type of their trajectories. **(B)** Average relative abundance of bacterial families for infants within the same trajectory. Families with below 10% of relative abundance and prevalence are summed and grouped as “Other.” **(C)** The proportion of infants born by CS and VD infants with or without IAP in each trajectory.

We next investigated the temporal stability of the identified clusters in individual infants. For this, we focused on a subset of 46 infants for which all four samples were available. Interestingly, 54% of infants belonging to one of the two clusters, either *Bacteroidaceae-* or *Clostridiaceae-*driven cluster at 3 weeks, stayed in the same cluster throughout all the time points, suggesting that the initial microbiota composition after birth has a critical effect on future microbiota developmental trajectory until 1 year (Figure 3A). Consequently, two stable microbiota development trajectories and an unstable trajectory emerged. One microbiota developmental trajectory was followed by 19 (41%) infants, all VD, and was characterized by a high relative abundance of the family *Bacteroidaceae* (mean > 25% at all time points, IQR = 36%). Another microbiota trajectory was followed by 6 (13%) infants, with 50% CS infants and 50% VD infants, and was characterized by a virtual lack of *Bacteroidaceae* at all the time points (mean < 0.05% at all time points, IQR = 2%) and higher relative abundance of *Clostridiaceae*. Finally, the temporally instable intermediary trajectory was followed by 21 (46%) infants, with 48% CS infants and 52% VD infants (Figures 3B,C). The relative abundance of *Bacteroidaceae* was significantly different between all the trajectories, and *Clostridiaceae* was significantly different in the second and third trajectories compared to the first one (Pair-wise Wilcoxon, FDR-adjusted, *p* < 0.05). Interestingly, the abundance of *Bifidobacteriaceae, Enterobacteriaceae, Lachnospiraceae, Oscillospiraceae*, and *Veillonellaceae* did not differ consistently between the trajectories (Pair-wise Wilcoxon, FDR-adjusted, *p* > 0.05). Additionally, the alpha diversity (Shannon diversity index) was similar for all the trajectories at all the time points (Pair-wise Wilcoxon, FDR-adjusted, *p* > 0.05, on Shannon diversity index). We compared the trajectories in terms of stability of bacterial composition by calculating the presence-absence Jaccard index of the genus-level composition for successive time points for each child. As expected, the “intermediary” trajectory presented a less stable composition over time, with successive communities for each infant being more dissimilar than for the first and second trajectory (pair-wise Wilcoxon, FDR-adjusted, *p* < 0.05 on presence/absence Jaccard distance). The trajectories were strongly associated with delivery mode (Fisher test, FDR-adjusted, *p* < 0.1) but not with IAP exposure (Fisher test, FDR-adjusted, *p* > 0.1 between VD and VD + IAP infants). The trajectories were also not significantly associated with the number of antibiotic courses received by the infants during the first year of life (Fisher's exact test, FDR-adjusted, *p* > 0.1). Additionally, starting age of solid food and breastfeeding habits (exclusive breastfeeding and breastfeeding period length) were not significantly different between the three trajectories (Fisher's exact test, FDR-adjusted, *p* > 0.1). All the comparisons between trajectories are available in Supplementary File 5.

### CS-like *Bacteroides* depletion in early life microbiota can be observed in a subset of VD infants

**Figure 4:**
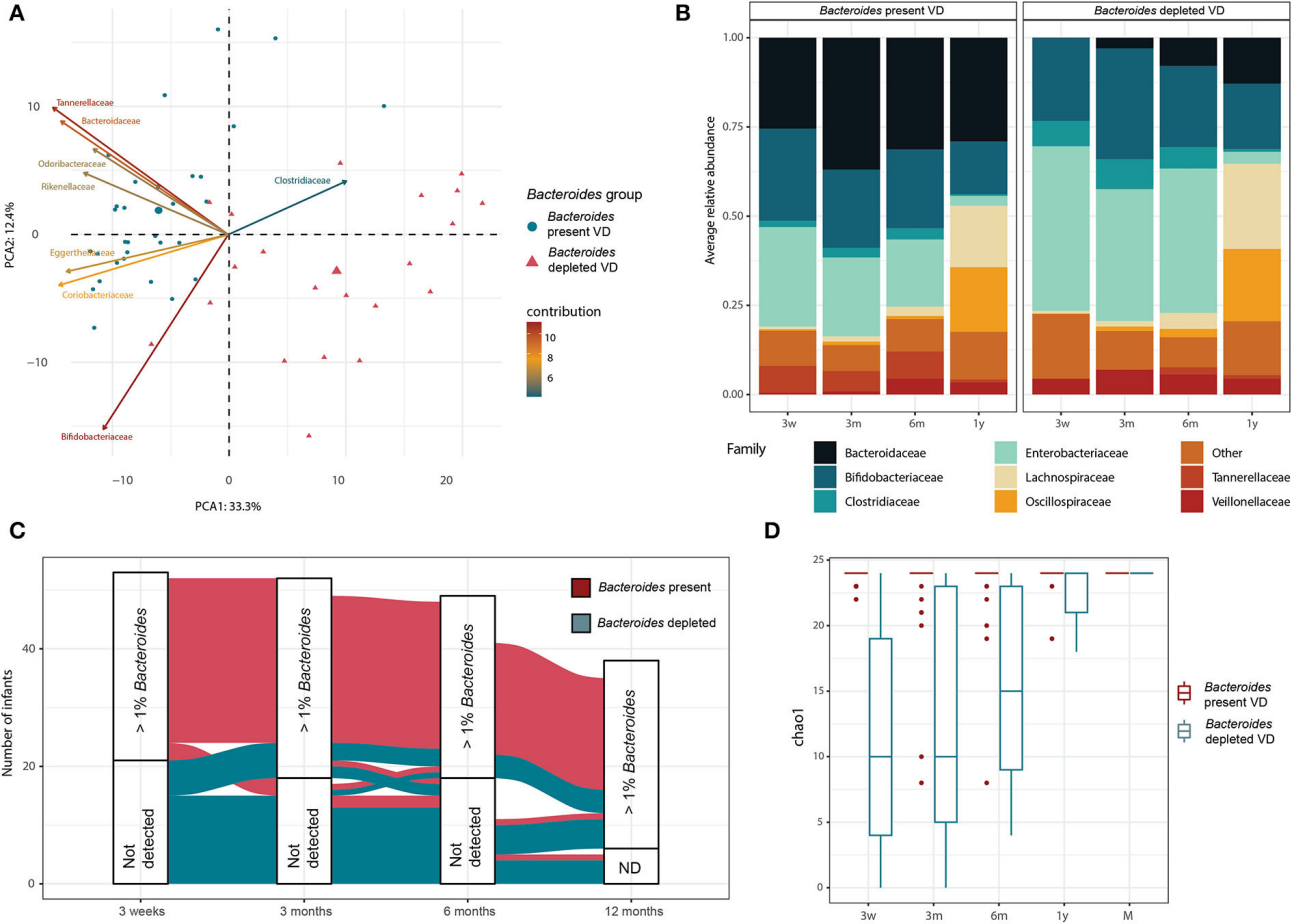
Depletion of genus *Bacteroides* in a subset of VD infants. **(A)** PCA biplot based on family-level taxonomic profiles for VD infants at 3 weeks of age. Taxonomic profiles obtained for the 3-week samples were aggregated at the family level, and low abundance taxa were excluded before computing an Aitchinson distance between samples. Samples in the PCA biplot are colored as per the groups based on relative abundance of *Bacteroides* genus. Bacterial families contributing most to the observed variation were plotted as arrows on the biplot. **(B)** Average microbiota composition for infants grouped by the abundance of *Bacteroides* at 3 weeks. Taxonomic profiles are aggregated at the family level, and families with below 10% of relative abundance and prevalence are summed and grouped as “Other.” **(C)** Alluvial plot showing the evolution over the first year of life of infants according to the relative abundance of *Bacteroides*. A cutoff of 1% relative abundance of *Bacteroides* was chosen to group the samples, and the infants are colored by the relative abundance of *Bacteroides* at 3 weeks. **(D)** The taxonomic richness of the *Bacteroides* species (Chao1 index) in the infants and their respective mothers according to *Bacteroides* relative abundance at 3 weeks.

We further investigated the abundance and dynamics of the genus *Bacteroides* that was driving the clustering and trajectories. At 3 weeks, the microbiota of all the CSD infants contained a low relative abundance of the genus *Bacteroides* (mean = 0.2% relative abundance, IQR = 0.2%). At 3 weeks, we had 53 VD infants as opposed to 55, since samples from two infants at this time point were not available. Strikingly, roughly 40% (*n* = 21) of the VD infants (*n* = 10 in group VD with IAP, *n* = 11 in group VD without IAP) presented an equivalently low relative abundance (below 1%) of the genus *Bacteroides* at 3 weeks. To explore this unusual microbiota composition for the VD infants, we defined the two groups of VD infants according to the relative abundance of the genus *Bacteroides* at the first sampling point, i.e., at the age of 3 weeks. A group of 21 infants, called hereafter “*Bacteroides-*depleted VD” had a < 1% relative abundance of *Bacteroides* in their microbiota. The rest of the VD infants (*n* = 32) were called “*Bacteroides-*present VD.” The “*Bacteroides* VD” groups were consistent with the trajectories defined previously, with 90% of the “*Bacteroides*-present VD” infants following the first trajectory and 92% of the “*Bacteroides*-depleted VD” infants following the second or intermediary trajectory (Supplementary File 6). The global bacterial composition of the two groups was significantly distinct (PERMANOVA on family-level Aitchinson distance, *p* = 0.001; 999 permutations) at 3 weeks (Figure 4A). The two groups were also distinct in terms of their functional composition (PERMANOVA on KO Bray-Curtis distance, *p* = 0.001; 999 permutations). Altogether, 62 MetaCyc prokaryotic pathways were significantly differentially abundant in the two VD infant groups at 3 weeks (linear model, *q* < 0.1), in a large part, overlapping with the pathways identified as differentially abundant in clusters 3w-1 and 3w-2 (Supplementary File 2).

When comparing the microbiota developmental trajectories of the two groups of VD infants, the “*Bacteroides-*depleted VD” infants showed a gradual acquisition of *Bacteroides* during the first year of life (Figure 4B). Yet, while strongly depleted in *Bacteroides* at 3 weeks, 75% of the “*Bacteroides-*depleted VD” infants had a *Bacteroides* relative abundance above 1% at 12 months (average 13%). On the other hand, only three infants from the “*Bacteroides-*present group” showed a loss in *Bacteroides* relative abundance of < 1% at any time point, while the remaining infants for that group had a *Bacteroides* relative abundance above 1% at all the sampling time points (Figure 4C). Globally, the richness of the *Bacteroides* species increased during growth, while the “*Bacteroides-*depleted VD” infant group had significantly lower *Bacteroides* richness at all the time points (Wilcoxon *p* < 0.05) (Figure 4D). A total of 24 species of *Bacteroides* were detected in the dataset, with *B. fragilis, B. dorei*, and *B. vulgatus* being the most abundant. However, no *Bacteroides* species were found to be present in one only infant group, indicating differences in the abundances of *Bacteroides* species but not in their prevalence (Supplementary Figure 7). Taken together, these results suggest that the *Bacteroides* depletion observed in the “*Bacteroides-*depleted VD” infant group at 3 weeks is general rather than a lack of specific species of *Bacteroides*, and has a long-term effect that impacts the microbiota development of the infants during the first year of life.

To explore the potential reasons for the depletion of *Bacteroides* in VD infants, we compared the prenatal and perinatal variables between the “*Bacteroides-*present VD” and “*Bacteroides-*depleted VD” infants. We first explored birth-related differences between the two groups, such as potential IAP exposure, intensive care, the time between water breakage and delivery, gestational age, and birth weight. None of these variables were significantly associated with the two infant groups. Next, we explored maternal variables such as the mother's age, maternal health during pregnancy, exposure to antibiotics or probiotics during pregnancy, number of prior pregnancies, and pre-pregnancy BMI. Once more, none of these variables were significantly associated with the two infant groups. We also tested against group membership the antibiotics received by the children in the first 3 weeks of life and breastfeeding habits; but once again, no significant association could be found. A complete list of the variables and their definition, and the result of the statistical comparison are available in Supplementary File 3.

We next compared the composition of the maternal gut microbiota in the samples collected prior to delivery for the two VD groups (Supplementary Figure 8), and in particular in the *Bacteroides* species (Supplementary Figure 7). Overall, no differences were observed between the microbiota composition of the mothers of “*Bacteroides-*present VD” and “*Bacteroides-*depleted VD” infants at the family level (PERMANOVA on family-level Aitchinson distance, *p* = 0.26; 999 permutations), and we could not identify any significantly differentially abundant taxa between the two groups of mothers using MaAsLin2 at the family and species levels (negative binomial, *q* > 0.1). In order to rule out any effect of technical variations, we explored the association between the two groups of infants with the length of sample storage, the person performing DNA extraction, DNA extraction yield, and sequencing depth. Interestingly, DNA yield (ng of DNA per gram of feces) was significantly higher in the “*Bacteroides-*present VD” than in the “*Bacteroides-*depleted VD” (FDR-adjusted *p* < 0.1, Wilcoxon test). Similarly, the DNA yield obtained from 3 weeks samples of CSD infants was significantly lower (median 1,762 ng/g of feces) compared to the “*Bacteroides*-present VD” (median 5,495 ng/g of feces) but was similar to the “*Bacteroides*-depleted VD” samples (median 1,575 ng/g of feces, FDR-adjusted *p* < 0.1 Wilcoxon test). Importantly, the lack of significant associations between birth and maternal variables and the “*Bacteroides-*depleted VD” group could be due to our limited cohort sampling size. Indeed, our study was powered to only reliably detect large effect sizes (d = 0.82 for the power of 0.8 by the Wilcoxon test).

## Discussion

Birth mode is a major factor influencing the development of infant gut microbiota and has anticipated effects on infants' immune systems and long-term health (Mueller et al., 2015; Korpela, 2021). More specifically, CSD infants have a characteristically reduced colonization and abundance of *Bacteroides* spp. (Dominguez-Bello et al., 2010; Bokulich et al., 2016; Wampach et al., 2018; Guittar et al., 2019; Reyman et al., 2019; Busi et al., 2021). In this study, we leveraged the shotgun metagenomics approach on 78 infants from a well-characterized longitudinal birth cohort followed up from 3 weeks to 1 year of age. Our results recapitulate the previous findings that show a strong influence of birth mode on infant microbiota acquisition. Apart from comparing the infant microbiota composition and function based on birth groups, we zoomed into data-driven infant microbiota clusters and trajectories. Our results show a strong depletion of genus *Bacteroides* in 40% of the VD infants. This study expands our understanding of the impact of various early life factors on the colonization and dynamics of *Bacteroides* spp. in infants.

As previously reported in several longitudinal infant cohorts, we observed the maturation of the infant gut microbiota over the first year of life in terms of taxonomic composition and diversity, with an increase in taxonomic alpha diversity and richness especially observed around the age of weaning (Bäckhed et al., 2015; Beller et al., 2021). Consistent with previous studies (Yatsunenko et al., 2012; Bäckhed et al., 2015), the compositional microbiota maturation during the first year of life resulted in reduced beta diversity and global convergence in the gene content of the infant gut microbiota toward an adult-like composition. At the age of 1 year, the taxonomic composition of the infant gut microbiota was still distinct from that of the adult gut microbiota. Interestingly and contrary to the taxonomic maturation, the functional microbiota maturation was not characterized by an increase in gene family richness. This result also observed in previous studies (Yatsunenko et al., 2012; Wang et al., 2021), is likely due to a smaller fraction of sequences with assignable KEGG annotations, in particular in the adult samples, as a result of increased complexity. We did not observe any significant differences in the abundance of broad functional categories between the infant and mother samples or in the infant samples across the different time points. As reported previously (Wang et al., 2021), unlike taxonomic signatures, there is a high similarity between the microbial metabolic functions between infants and adults, underlining the importance of conserved core microbial functions.

We used a clustering approach to identify the main microbiota community type characteristics for each sampling time point. We applied hierarchical clustering on the Aitchinson distance, which allows us to take into account the compositionality of microbiome data and reduces effects due to sequencing depth differences between samples (Galloway-Pena et al., 2017). This method allowed us to identify two main infant microbiota clusters at each time point, driven in part by the relative abundance of the *Bacteroidaceae* family. Using cut-offs different from those in our study, Eck et al. reported comparable patterns, in particular describing two distinct infant gut microbiota settler types based on a cut-off of 30% of relative abundance of *Bacteroidetes* (Eck et al., 2020). A large meta-analysis covering > 10,000 microbiota samples from 17 countries and derived from children sampled between birth and 3 years identified four robust infant enterotypes typified by *Firmicutes, Bifidobacterium, Bacteroides*, and *Prevotella* (Xiao et al., 2021). While age was the strongest predictor of enterotype and all enterotypes included children from both birth modes, interestingly, the *Bacteroides*-dominated enterotype was detected in Northern European countries such as Finland, Norway, and Estonia in most sampling months. Hence, our results on Finnish infants may not be generalized across populations. In any case, many previous studies using the clustering approach have also reported the importance of *Bacteroidaceae* in infant or child gut microbiota datasets and its associations to geography, breastfeeding duration, butyrate synthesis, birth mode, human milk oligosaccharide (HMO) metabolizing capacity, orthogonal or collateral relationship with clusters driven by other taxa, and developmental stages (Yatsunenko et al., 2012; Stewart et al., 2018; Zhong et al., 2019; Berger et al., 2020; Casaburi et al., 2021; Roswall et al., 2021). Importantly, we observed the infant microbiota clusters to be associated with differences in several functional pathways, including pathways implicated in carbohydrate metabolism. In particular, degradation pathways of two glycans, β-mannans, and chondroitin sulfate were significantly associated with the *Bacteroidaceae-*rich cluster at all the time points. Chondroitin sulfate is a glycosaminoglycan prevalent in human milk and is associated with anti-inflammatory properties and protective effects in neonates (Knowles et al., 2021); it can be degraded by *Bacteroides* species (Shang et al., 2016). β-Mannans are complex plant-based sugars widespread in the human diet and can be degraded and used by *Bacteroides* species apart from some *Firmicutes* species (la Rosa et al., 2019). Overall, the results suggest that depletion in *Bacteroidaceae* contributes to a significant alteration in the ability of an infant's microbiota to metabolize complex sugars (Marcobal et al., 2011; Bäckhed et al., 2015; Casaburi et al., 2021). Our results expand on previous reports that have addressed the functional differences of infant gut microbiota in relation to CS birth and reduced abundance of *Bacteroides*. A previous study has reported that during the first 3 months of life, CSD infants have underrepresented biosynthetic pathways and that the vast majority of which (14/20) could be assigned to *Bacteroides* spp. (Wilson et al., 2021). A recent meta-analysis on infant metagenomes reported functions such as starch degradation, glycolysis, and queuosine biosynthesis to be enriched in a *Bacteroides-*dominated community type, which comprised 90% of VD infants (Xiao et al., 2021). In a recent randomized controlled trial, functional differences between CSD and VD infants were investigated on days 7 and 27, and the results indicated that 133 and 663 functional genes differed between the two birth groups, respectively (T. Dierikx et al., 2022). Wang et al. have also reported differences between metabolic functions in relation to birth mode, with functions such as vitamin, sugar, and cell wall biosynthesis higher in VD infants (Wang et al., 2021).

Strikingly, considering the infants that had samples available at all the time points, 54% of the infants clustering together at 3 weeks clustered together throughout all the sampling time points. This indicates the lasting influence of the pioneering community assemblance and aligns with previous reports about deterministic and largely predictable transition patterns between infant gut microbiota community types (Stewart et al., 2018; Xiao et al., 2021). The fact that more than half of the infants retained their characteristic high or low abundance of *Bacteroidaceae* allowed us to define three infant microbiota trajectories strongly associated with the birth mode. As expected, the “high *Bacteroidaceae* trajectory” was only observed in the VD infants, while the “low *Bacteroidaceae* trajectory” and “intermediary trajectory,” characterized by complete or milder depletion in *Bacteroidaceae* until 1 year of age, respectively, were observed not only in the CS born infants but also in the VD infants. While this phenomenon has not been well-discussed in the scientific literature, we are not the first to report it. Our results confirm previous observations that *Bacteroides* depletion is not only present in CS-born infants but can also be observed in 20–49% of VD infants (Yassour et al., 2016; Shao et al., 2019; Wilson et al., 2021). We further explored the plausible causes of *Bacteroides* depletion in VD infants. The depletion was prolonged and was only partially resolved after 1 year. While many studies (e.g., Shao et al., 2019) and a systematic review (T. H. Dierikx et al., 2020) have documented that intrapartum antibiotic exposures lead to depletion of *Bacteroides* in VD infants, we did not confirm such an association in our cohort. Similarly, Stearns et al. (2017) did not find IAP to decrease the relative abundance of *Bacteroides* among VD infants at 12 weeks, and a clinical trial where no VD infants were exposed to IAP found that 20% of their VD samples had a low *Bacteroides* signature (Wilson et al., 2021). Also, Yassour et al. observed a similar *Bacteroides* depletion profile in seven Finnish VD infants; however, with such a small subset of infants, they were unable to search for significant correlations with any clinical variables such as IAP (Yassour et al., 2016). Our results from the same cohort using absolute abundance estimates from 16S rRNA gene amplicon data (Jokela et al., In Press), as well as earlier targeted qPCR studies, identified no statistically significant differences in the absolute abundance of the *Bacteroides fragilis* group in VD infants in relation to IAP exposure (Aloisio et al., 2014; Corvaglia et al., 2016). In this study, the incidental courses of postnatal antibiotics did not explain the microbiota clustering or trajectories at any time point.

Despite extensive exposure and other metadata available for the study infants, we were not able to associate the differences in the relative abundance of *Bacteroides* in VD infants to any prenatal variable such as the mother's pre-pregnancy BMI or parity, or to perinatal and postnatal factors such as gestational age, stay in intensive care, time between water break and delivery, and infant weight at birth. Importantly, all the infants in our study were partially or exclusively breastfed until 3 months, with the majority of infants breastfed until 6 months, and no association was found between breastfeeding habits and the *Bacteroides* depletion observed. Similarly, an earlier study found *Bacteroides* depletion within the first 3months of life not to be related to feeding mode or antibiotic exposure after birth (Wilson et al., 2021). We also comprehensively explored the possible effect of technical variations such as time of storage of samples before processing, extraction date, and sequencing run. Interestingly, we found DNA extraction yield per gram of feces to be significantly reduced in the *Bacteroides-*depleted VD infants. While the relative abundance of this genus is known to be sensitive to sample storage and DNA extraction methods (Salonen et al., 2010; Bahl et al., 2012; Rinninella et al., 2019; Zhang et al., 2021), in our study, all the samples were frozen without delays with stabilization buffers and were extracted using the same DNA extraction protocol. Also, the sequencing libraries were normalized for the input DNA. Hence, unlike across different studies, we believe that within this study, the link between DNA yield and community composition is a biological effect rather than a technical effect. As in the fecal samples, the majority of the extracted DNA is from bacteria (Qin et al., 2010; Li et al., 2014; Sender et al., 2016; Matijašić et al., 2020), and our findings suggest that infants with *Bacteroides-*depleted community type may have a lower absolute abundance of bacteria in their stools. While outside of the scope of this study, this hypothesis could be explored by qPCR, potentially in conjunction with sequencing, to convert the relative abundance of microbiota profiles into estimates of absolute abundances (Jian et al., 2020).

Several studies have documented the prominent transmission of *Bacteroides* spp. from mothers to infants (Ferretti et al., 2018; Korpela et al., 2018, 2020; Wampach et al., 2018; Shao et al., 2019; Mitchell et al., 2020). Our team has also proven experimentally that maternal fecal microbiota transplant immediately after birth restores the levels of *Bacteroides* in CSD infants (Korpela et al., 2020). Strain-level metagenome studies have shown that maternal strains are more likely to persist than other bacteria and that the retention of *Bacteroides* is higher in the infant's gut compared to other genera, terming them as “persisters” (Ferretti et al., 2018; Korpela et al., 2018; Lou et al., 2021; Wang et al., 2021). To investigate a possible role of the mother-infant transmission of *Bacteroides* in the present or depleted-Bacteroides groups, we studied the effect of maternal fecal microbiota composition. Fecal samples were collected from the mothers in the 2 weeks preceding delivery, and no differences in terms of fecal microbiota composition, either overall or within the composition or abundance of *Bacteroides* spp., could be detected between the two groups of mothers.

## Conclusion

Our study explored the gut microbiota developmental trajectories in healthy infants from 3 weeks to 1 year of age. Our results confirm previous reports indicating depletion of *Bacteroides* in CSD infants but also in a significant proportion of VD infants. Despite screening of extensive metadata, the cause of the depletion in VD infants still remains unresolved and would require larger infant cohorts where particularly the effect of different breastfeeding patterns could be investigated.

## Data availability statement

The sequence data that support the findings of this study are available under the BioProject ID: PRJEB52774. The names of the repository/repositories and accession number(s) can be found in the article/Supplementary material.

## Ethics statement

The studies involving human participants were reviewed and approved by the Ethical Committee of the Hospital District of Helsinki and Uusimaa. Written informed consent to participate in this study was provided by the participants' legal guardian/next of kin.

## Author contributions

AS, KK, K-LK, and WMdV designed and established the cohort. AS and WMdV managed the cohort. ED performed the sample collection, management, and DNA extraction. DM, AP, and AS planned and carried out the computational experiments, analyzed the data, and wrote the manuscript. All authors contributed to the manuscript revision, read, and approved the submitted version.

## Funding

This study was supported by grants from Tekes 329/31/2015 (WMdV), European Union's Horizon 2020 Research and Innovation Program H2020 MSCA (Sweet Crosstalk) project under grant agreement No. 814102 (AS), Academy of Finland 1325103 (AS), and 339172 (AP), Mary and Georg C. Ehrnrooth Foundation (AS), and Päivikki and Sakari Sohlberg foundation (AS). DM acknowledges the funding for Ph.D. received through European Union's H2020-MSCA-ITN-2018 Sweet Crosstalk project under grant agreement No. 814102.

## Conflict of interest

The authors declare that the research was conducted in the absence of any commercial or financial relationships that could be construed as a potential conflict of interest.

## Publisher's note

All claims expressed in this article are solely those of the authors and do not necessarily represent those of their affiliated organizations, or those of the publisher, the editors and the reviewers. Any product that may be evaluated in this article, or claim that may be made by its manufacturer, is not guaranteed or endorsed by the publisher.

## Abbreviations

VD: Vaginally delivered
CSD: Cesarean-section delivered
CS: Cesarean section
HMOs: Human milk oligosaccharides
IAP: Intra-partum antibiotic prophylaxis
HELMi: Health and Early Life Microbiota
RBB: Repeated bead beating
FDR: False discovery rate
PERMANOVA: Permutational multivariate analysis of variance
ANOVA: Analysis of variance
PCoA: Principal coordinate analysis
qPCR: Quantitative polymerase chain reaction.
