# Supplementary material for: Bacteroides abundance drives birth mode dependent infant gut microbiota developmental trajectories
Source: Front Microbiol. 2022 Oct 6;13:953475. doi: 10.3389/fmicb.2022.953475 (PMC9583133; doi:10.3389/fmicb.2022.953475)
Supplement: Supplementary file 7 [file Data_Sheet_7.DOCX]

Supplementary Material

**
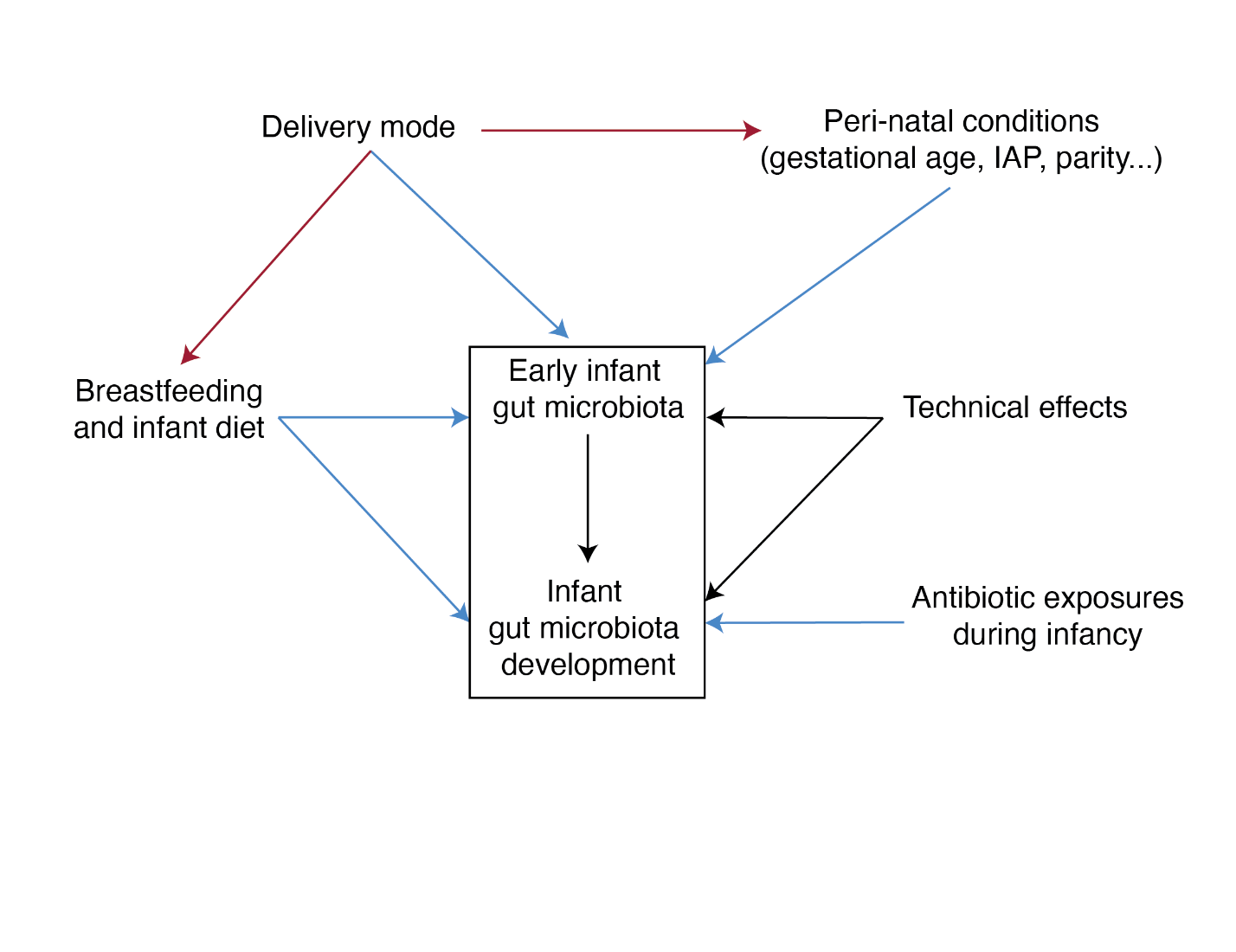
**

**Supplemental Figure 1.**  Directed acyclic graph (DAG) showing the causal relationships affecting the association between early-life exposures to infant gut microbiome. Arrows in blue represents theoretical associations of interest. Arrows in red represent potential confounding interactions between exposures.


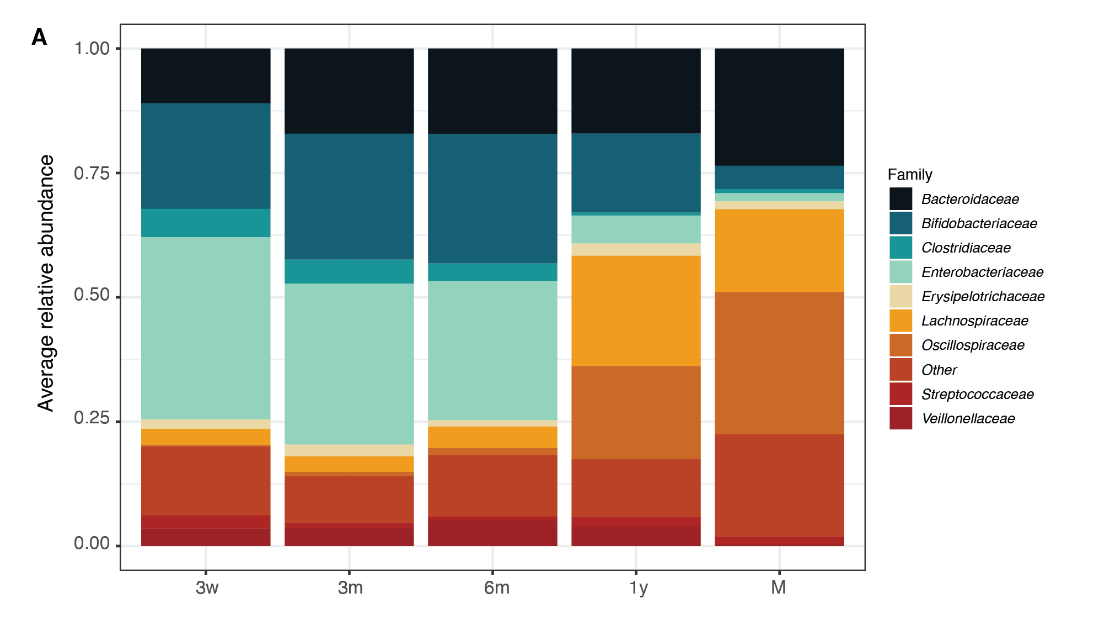


**Supplemental Figure 2.** Braken taxonomic profiling aggregated at the family level
Taxa representing less than 10% abundances and prevalence were summed and grouped as “Other”.


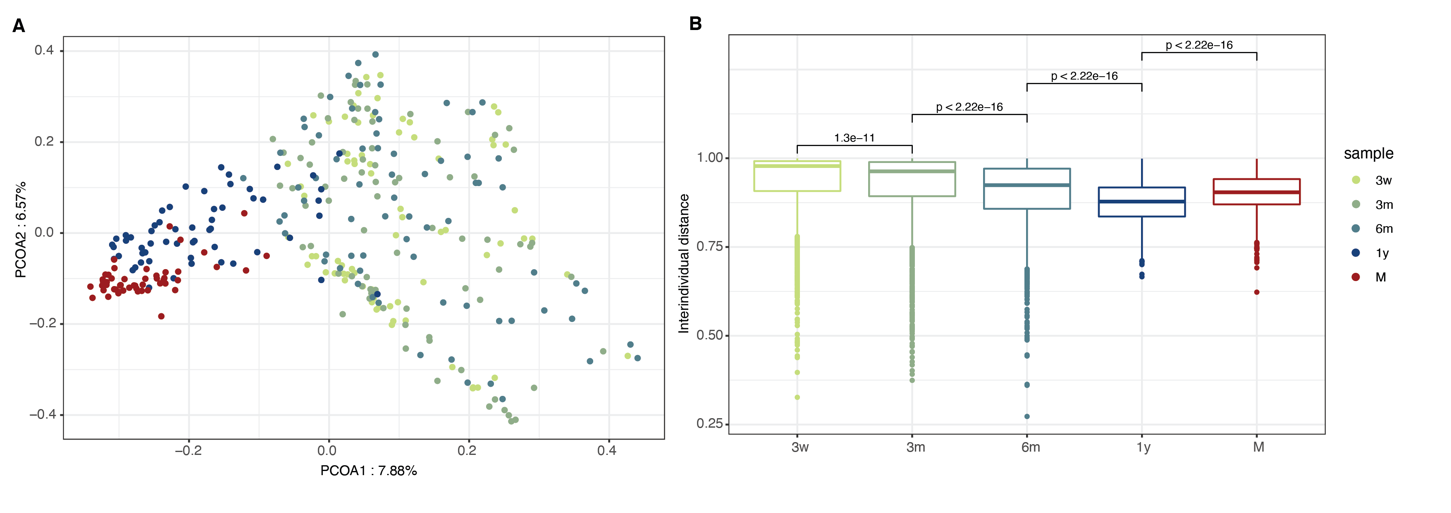


**Supplemental Figure 3.** Maturation of the microbiota during the first year of life on k-mer sequence composition (A) PCoA on Bray-Curtis distance on sample k-mer composition (B) Interindividual Bray-Curtis distances on sample k-mer composition


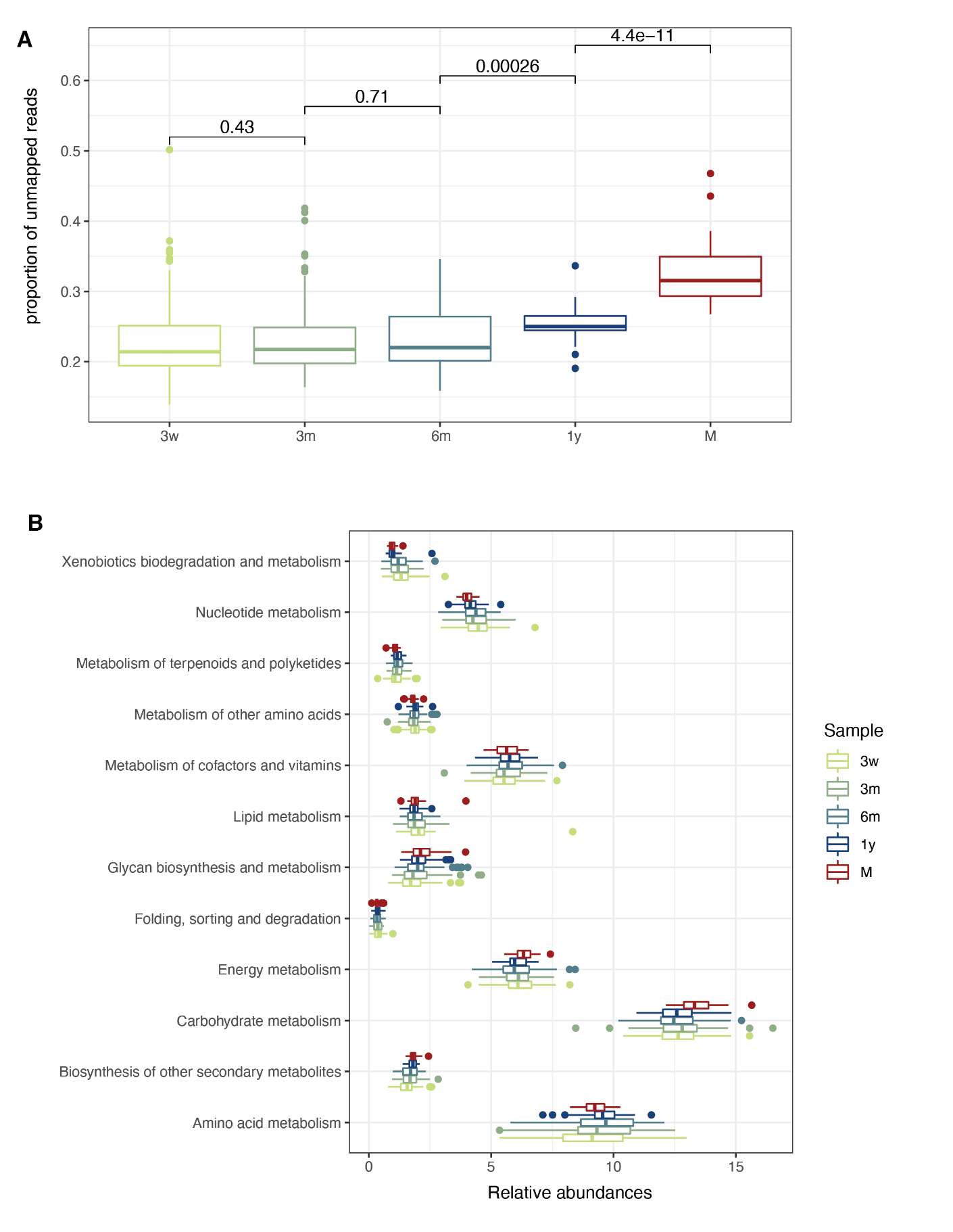


**Supplemental Figure 4.** (A) Percentage of metagenomic reads that failed to be annotated in a gene family by time point (B) Relative abundance of KEGG metabolic pathways of the microbiome at different ages.


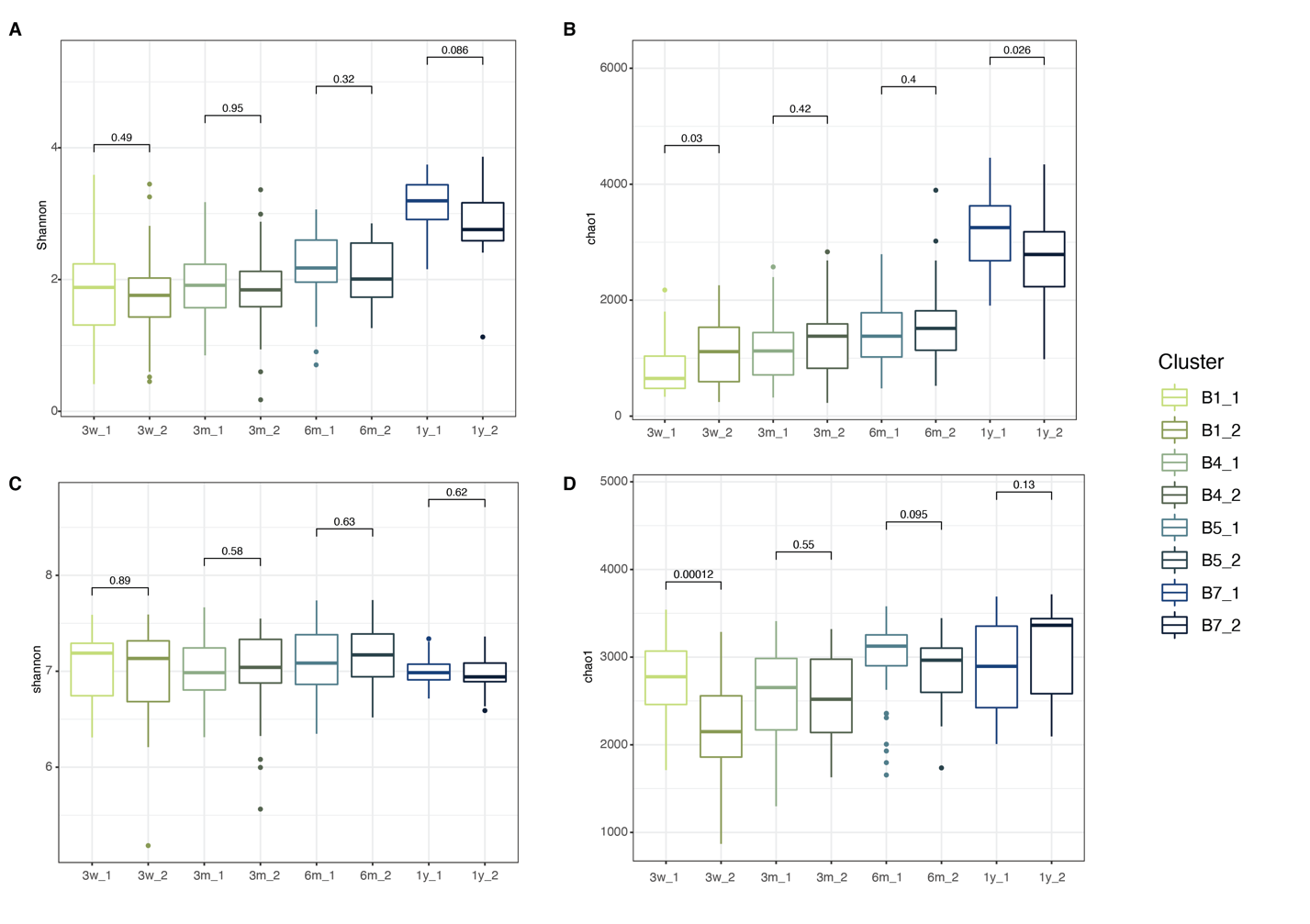


**Supplemental Figure 5.** (A) Taxonomic Alpha-diversity (Shannon diversity index), (B) taxonomic richness (Chao1 index) between the clusters and (C) Functional alpha-diversity (Shannon diversity index), (D) functional richness (Chao1 index)


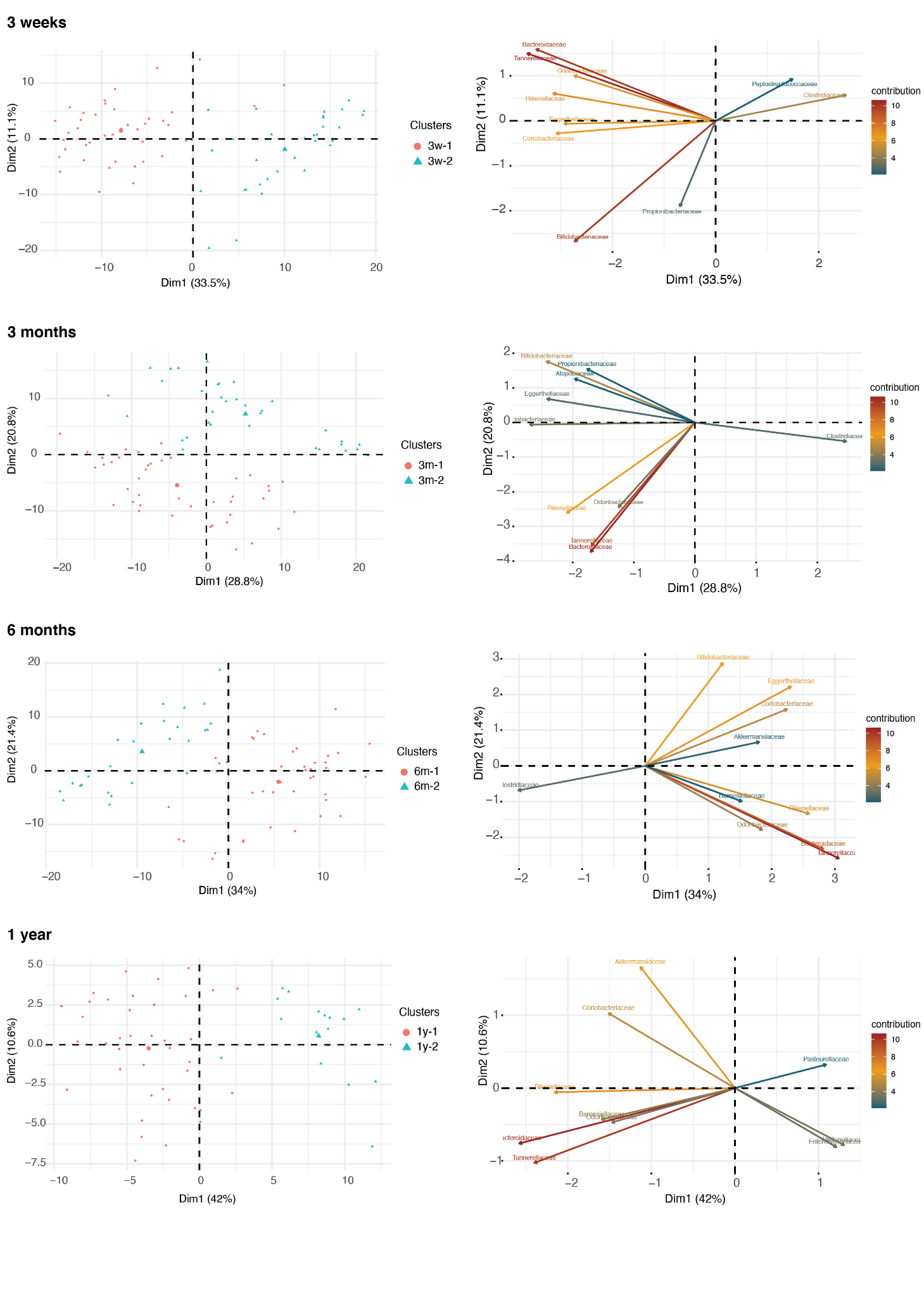


**Supplemental Figure 6.** PCA biplot at each time point


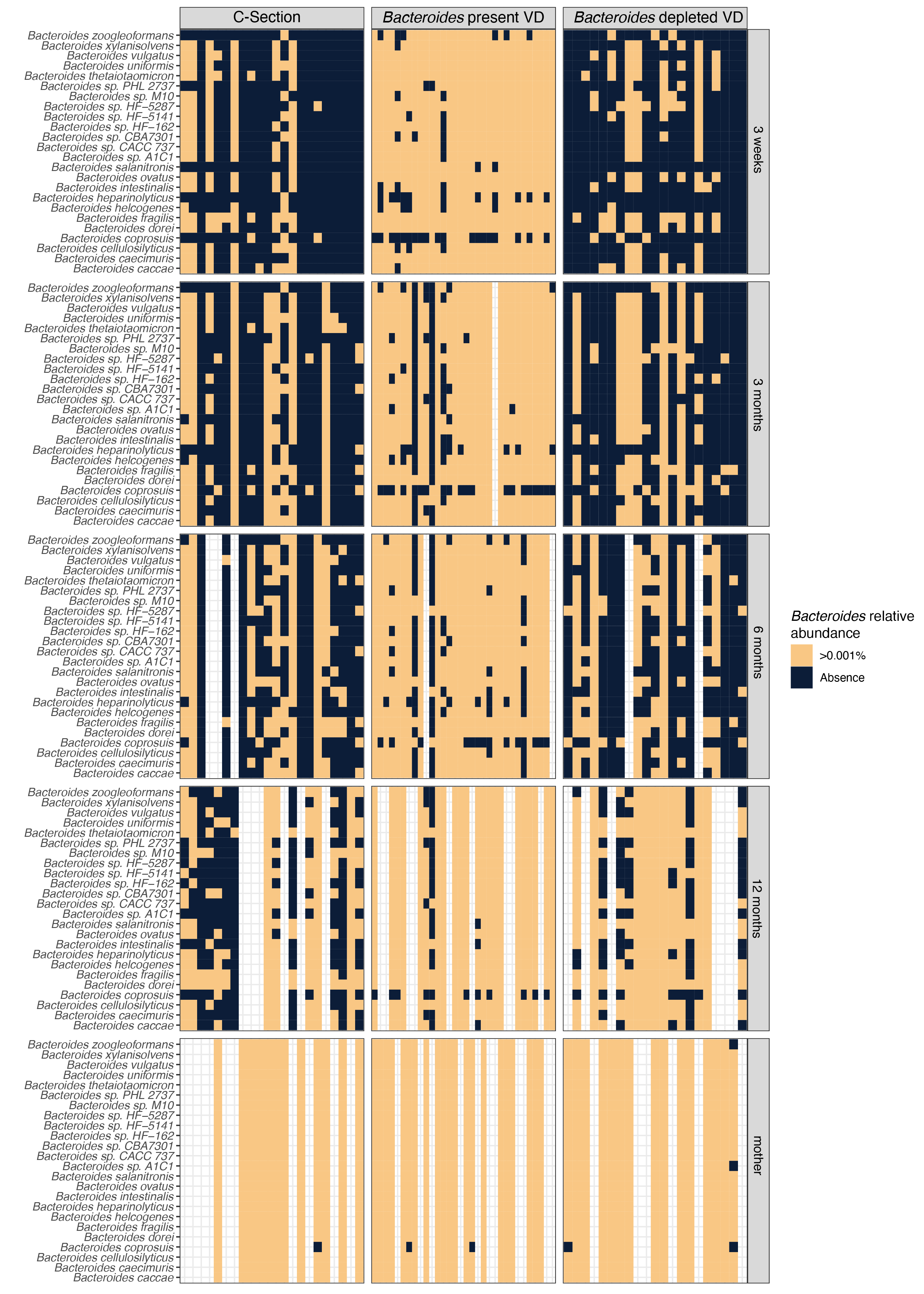


**Supplemental Figure 7.** Tile plot comparing the presence/absence of Bacteroides species in CSD infants, ‘*Bacteroides* present VD’ and ‘*Bacteroides* depleted VD’ infants at different time point and the corresponding mother samples

Cutoff of 0.001% relative abundance was used to signify the presence of a species in the sample. Samples are organized in each group (C-Section, *Bacteroides* present VD and *Bacteroides* depleted VD) on the x-axis according to their similarity in content at the family level using Aitchison distance and ward clustering.


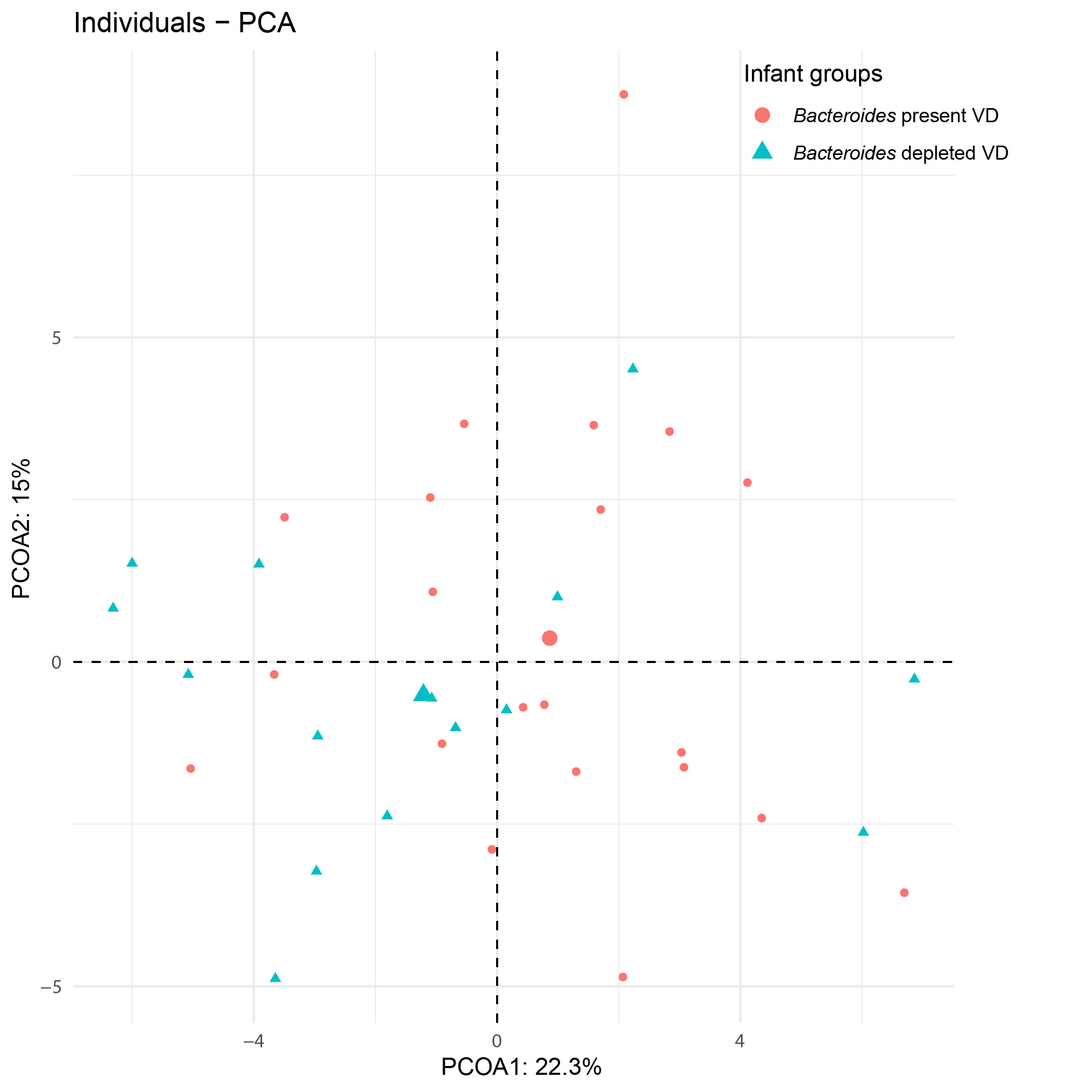


**Supplemental Figure 8.** PCA on Aitchinson distance at Family level - Mother samples at colored by their corresponding infant VD *Bacteroides* groups

| **groups** | **CSD** | **VD** | **VD with IAP** | **p value** |
| --- | --- | --- | --- | --- |
| n (%) | 23 | 32 | 23 |  |
| gender female, n (%) | 12 (52.1) | 18 (56.2) | 8 (34.7) | 0.27 |
| Mother BMI pre-pregnancy, mean (SD) | 23.73 (3.21) | 23.83 (3.91) | 22.52 (2.17) | 0.30 |
| Gestational age in weeks, median (IQR) | 39.71 (1.78) | 40.57 (1.50) | 40.43 (0.79) | 0.24 |
| Antibiotic treatments during pregnancy n (%) | 5 (21.7) | 6 (18.8) | 8 (34.8) | 0.44 |
| Birth weight in grams, mean (SD) | 3532.78 (455.28) | 3522.03 (541.54) | 3589.47 (361.84) | 0.86 |
| Ruptured membranes in hours, mean (SD) | 5.55 (11.94) | 7.93 (9.20) | 13.49 (13.92) | 0.08 |
| First skin contact in hours, mean (SD) | 1.21 (3.98) | 0.37 (2.12) | 0.52 (2.50) | 0.55 |
| Has siblings, n (%) | 4 (17.3) | 17 (53.1) | 6 (26) | **0.01** |
| Household has pets, n (%) | 7 (30.4) | 17 (53.1) | 3 (13) | **0.01** |
| Parents’ education score, median (IQR) | 2 (1) | 2 (0.25) | 2 (0.5) | 0.47 |
| Any breastfeeding >9 months, n (%) | 20 (86.9) | 30 (93.7) | 20 (86.9) | 0.40 |
| Exclusive breastfeeding until 3 months, n (%) | 12 (52.1) | 23 (71.8) | 13 (56.5) | 0.37 |
| Age of starting solid food in weeks, median (IQR) | 22 (7.5) | 24 (6.5) | 20 (3) | 0.33 |
| Has received antibiotic treatments during the 1st year of life, n (%) | 1 (4.3) | 11 (34.3) | 5 (21.7) | **0.02** |
| Number of samples, 4 samples, n (%) | 13 (56.5) | 21 (65.6) | 12 (52.1) | 0.58 |

**Table 1**. Cohort baseline characteristics*.* Background and early exposures characteristics were stratified by mode of delivery. Continuous and normally distributed variables are shown as means with standard deviations (SD) and an ANOVA test was used to compare the birth groups. Continuous but non-normally distributed variables are shown as medians with interquartile ranges (IQR), and the birth groups were compared using Kruskall-Wallis test. Finally categorical variables are shown in absolute numbers with percentages (%) and significant differences between categorical variables were tested with chi-square tests.
The p-values of variables that differed significantly between the birth groups are bold and italicized for clarity.
